# DNA Methylation Participates in Drought Stress Memory and Response to Drought in *Medicago ruthenica*

**DOI:** 10.3390/genes15101286

**Published:** 2024-09-30

**Authors:** Na Zi, Weibo Ren, Huiqin Guo, Feng Yuan, Yaling Liu, Ellen Fry

**Affiliations:** 1 Inner Mongolia Key Laboratory of Grassland Ecology, School of Ecology and Environment, Inner Mongolia University, Hohhot 010021, China; nazi9822@163.com; 2 School of Life Science, Inner Mongolia Agriculture University, Hohhot 010010, China; huiqinguo@126.com; 3 Key Laboratory of Forage Breeding and Seed Production of Inner Mongolia, National Center of Pratacultural Technology Innovation, Hohhot 010010, China; mengcaoyf666@163.com (F.Y.); liuyaling0719@163.com (Y.L.); 4 Department of Biology, Edge Hill University, Ormskirk L39 4QP, UK; fryel@edgehill.ac.uk

**Keywords:** epigenetic, DNA methylation, stress memory, drought stress, *M. ruthenica*

## Abstract

**Background**: Drought is currently a global environmental problem, which inhibits plant growth and development and seriously restricts crop yields. Many plants exposed to drought stress can generate stress memory, which provides some advantages for resisting recurrent drought. DNA methylation is a mechanism involved in stress memory formation, and many plants can alter methylation levels to form stress memories; however, it remains unclear whether *Medicago ruthenica* exhibits drought stress memory, as the epigenetic molecular mechanisms underlying this process have not been described in this species. **Methods**: We conducted methylome and transcriptome sequencing to identify gene methylation and expression changes in plants with a history of two drought stress exposures. **Results**: Methylation analysis showed that drought stress resulted in an approximately 4.41% decrease in *M. ruthenica* genome methylation levels. The highest methylation levels were in CG dinucleotide contexts, followed by CHG contexts, with CHH contexts having the lowest levels. Analysis of associations between methylation and transcript levels showed that most DNA methylation was negatively correlated with gene expression except methylation within CHH motifs in gene promoter regions. Genes were divided into four categories according to the relationship between methylation and gene expression; the up-regulation of hypo-methylated gene expression accounted for the vast majority (692 genes) and included genes encoding factors key for abscisic acid (ABA) and proline synthesis. The hypo-methylation of the promoter and body regions of these two gene groups induced increased gene transcription levels. **Conclusions**: In conclusion, DNA methylation may contribute to drought stress memory formation and maintenance in *M. ruthenica* by increasing the transcription levels of genes key for ABA and proline biosynthesis.

## 1. Introduction

Climate drought is a phenomenon that can have negative impacts on the natural environment and human well-being, and it has become a focus of research attention in recent years, since drought events have increased [1,2]. Drought stress can impair plant growth and development, ultimately affecting crop productivity [3,4]. Hence, understanding the genetic and molecular mechanisms underpinning plant resistance to drought is critical [5]. Plants can undergo genetic or biochemical modifications when facing drought stress for the first time, which result in differences in their responses to future stress, which is a process referred to as drought stress memory [6]. Drought stress memory can be transmitted through mitosis, where plants that have been subjected to one drought will show stronger physiological responses when subjected to a subsequent drought than when they are subjected to drought treatment for the first time [7,8,9,10]. Transcription responses during repeated exposures to stress differ from those occurring during a single exposure [11]. Exposing plants to stress-induced hormone accumulation may alter their response to subsequent stresses, improving survival rates [12,13]. In addition to changes in physiological parameters, such as abscisic acid (ABA), after repeated exposure to stress, certain stress responsive genes exhibit transcriptional changes that differ significantly from their initial response [14,15]. This phenomenon occurs because plant genes exhibit transcriptional memory response patterns to drought stress, which can further alter cellular responses and crosstalk among molecular pathways by changing transcript or encoded protein levels [11,16,17]. To meet the definition of transcriptional memory, transcript levels produced under continuous dehydration stress reactions must differ from those in response to initial stress exposure [18]. Four types of dehydration stress memory genes have been identified in *Arabidopsis* spp. and *Zea mays*, and their transcriptional behavior under repeated stress is more complex than that in response to a single dehydration stress, mainly manifesting as differences in gene transcript levels during the first and last stress exposure [11,16]. Hence, drought stress memory involves epigenetic modification coordinated by multiple signaling pathways [19].

Numerous factors, including epigenetic modification, influence changes in transcription levels. Epigenetic changes can involve DNA methylation, histone modification, miRNA, and transcription factor modification, which may be among the molecular mechanisms underlying drought stress memory. These alterations can affect gene expression by influencing chromatin state without changing DNA sequence, and they are heritable with important roles in regulating plant growth and development. DNA methylation is a relatively stable modification, which participates in plant drought stress memory formation and maintenance [20,21]. Particular methylation types, or increased or decreased methylation levels, occur at specific DNA locations to influence stress response-related molecular pathways and increase related enzyme activity, thus protecting functional gene expression against the impact of stress to preserve plant growth and development [17,22,23].

*M. ruthenica* is an allogamous diploid (2*n* = 16) perennial leguminous forage grass with a plant height of 0.2–1 m and a creeping growth pattern; it is widely distributed in grasslands, sandy areas, riverbanks, and hillside wilderness with sandy soil in China, Mongolia, and Russia. The *M. ruthenica* distribution area is characterized by dry infertile soils and long cold winter times. *M. ruthenica* is a close relative of alfalfa (*Medicago sativa*), and genome and transcriptome sequencing indicate that it retains many genes involved in abiotic stress tolerance relative to other alfalfa varieties [5,24]. There is evidence that *M. ruthenica* has high tolerance to environmental stress and represents a precious genetic resource for understanding and improving tolerance to adversity [24,25,26]; hence, this species represents a valuable model for studies in legume grasses and particularly for understanding the molecular mechanisms underlying alfalfa tolerance to environmental stress. The expanded *FHY3*/*FAR1* family is involved in *M. ruthenica* tolerance to drought stress [24]; for example, the *C2H2* transcription factor family, which is an important regulator of drought and salt tolerance, is expanded to 203 members in the *M. ruthenica* genome compared with *M. sativa* [26]. Therefore, we chose *M. ruthenica* as the experimental material in this study. The investigation of epigenomic variations in plants has mainly been limited to a few model species, such as rice and *Arabidopsis*. To date, no research into the drought stress memory of *M. ruthenica*, based on genomic methylation modification, has been reported.

The purpose of our study was to explore the drought stress memory transcriptional responses of *M. ruthenica*, based on DNA methylation, and to investigate the following three issues in depth: (1) whether *M. ruthenica* develops stress memory at the gene transcription level after repeat drought stresses; (2) what effect drought stress has on genomic methylation; and (3) the relationship of methylation group modifications with changes in gene transcription.

## 2. Materials and Methods

### 2.1. Plant Material and Drought Treatments

**Figure 1:**
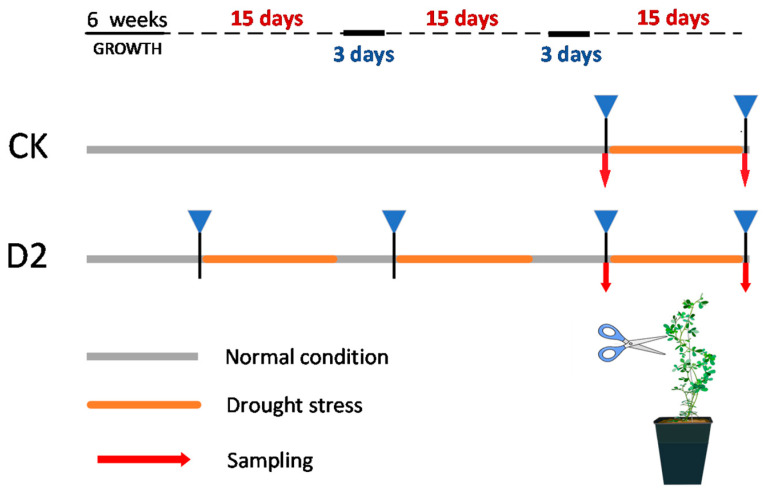
Drought stress treatment of *M. ruthenica* experimental groups in this study (CK: control; D2: stress memory).

The experimental material used in this study was a wild cultivated variety of *M. ruthenica* (L.) Sojakcv. Tumote, national approved variety, registration number 379; the plant materials used were identified by Zhaolan Wang. *M. ruthenica* seeds were scarified using sandpaper and then incubated in the dark at 4 °C for 3 days. Next, seeds were placed in a growth chamber (temperature, 25 °C ± 2 °C; photoperiod, 16 h light/8 h darkness; relative humidity, 50%). After germination, seeds were transferred to pots (8 × 8 × 10 cm) filled with nutrient soil (PINDSTRUP company) and vermiculite (1:1), with one plant in each pot, under the same growth conditions. Six-week-old seedlings were randomly divided into two groups: a control group with no stress history (CK) and another group with a history of two drought stress exposures (D2) (*n* = 45 per group). D2 seedlings were first subjected to drought stress twice; then, both groups were subsequently subjected to drought stress simultaneously. Drought treatment comprised 15 days in 30% soil moisture content. After exposure to drought stress, plants underwent a 3-day rehydration period, during which soil moisture content was maintained at >80%. Details of drought stress and water recovery treatment are presented in Figure 1. Plant phenotypic traits were observed after each stress and rehydration, and leaf samples were collected before and after the last stress exposure. Sampling times were before the last drought (DB) and after the last drought (DA). DB samples were used for the measurement of physiological parameters, whole genome bisulfite sequencing, and transcriptome sequencing, while DB and DA samples were used for real-time fluorescence quantification. For transcriptome and methylation sequencing, samples were collected from three plants (biological replicates) under each treatment condition, immediately frozen in liquid nitrogen, and then transferred to a −80 °C refrigerator for downstream processing.

### 2.2. Morphological Traits

*M. ruthenica* morphological traits, including height, stem diameter, leaf number, leaf area, internode length, total biomass, aboveground biomass, and underground biomass, were measured. Height was defined as absolute plant height, which was measured as the straightened length from the soil surface to the top of the leaves. Leaf area was measured using a leaf area meter (LI-3000 Portable Area Meter, LI-COR, Lincoln, NE, USA). The middle leaflet of the top three compound leaves of the plant was chosen for leaf area measurement; three duplicate leaves were measured and mean values were calculated. Using a vernier caliper, the stem thickness of the main base, and the length of each stem node at different positions on the branch, middle, and bottom were measured, and the mean value was taken as the mean internode length. For biomass measurement, individual plants were divided into aboveground and belowground parts; then, they were dried at 65 °C for 24 h and weighed (accuracy, 0.01).

### 2.3. Leaf Physiological Variable Measurement

Leaves were collected (weight, 0.2 g for each variable) from the same growth site, from seedlings under different treatment conditions, and stored in an ice box for physiological indicator measurement.

### 2.4. Soluble Sugar Content

Weighed samples (0.1–0.2 g) were added to 1 mL of distilled water and ground into a homogenate, poured into a covered centrifuge tube, transferred to a 95 °C water bath for 10 min, allowed to cool, and centrifuged (8000× *g*, 25 °C, 10 min), and supernatants transferred into 10 mL test tubes. Samples were then diluted to 10 mL with distilled water and shaken well for later use. A spectrophotometer was preheated for >30 min, the wavelength was adjusted to 620 nm, and absorbance values were measured.

### 2.5. Malondialdehyde (MDA) Content

The Plant MDA Assay Kit with TBA (Suzhou Comin Biotechnology Co., Ltd., Suzhou, China) was used to determine MDA content in plant leaves, following the manufacturer’s instructions.

### 2.6. Proline Content

A reagent kit (Suzhou Comin Biotechnology Co., Ltd., Suzhou, China) was used to determine plant leaf proline content, following the manufacturer’s instructions for the operation and calculation methods.

### 2.7. ABA Content

ABA content was determined following previous reports [27,28]. Briefly, plant leaf samples were ground into powder under liquid nitrogen; then, they were placed in 2 mL centrifuge tubes, and a methanol acetonitrile aqueous solution was added (40:40:20). Samples were shaken, mixed for 2 min, and then extracted at 4 °C for 12 h under light protection and centrifuged at 14,000 r/min for 10 min. The supernatant was taken and dried with nitrogen. A constant volume of methanol aqueous solution (50:50, *v*/*v*) was then added, which was followed by centrifugation (10 min, 14,000 r/min) and analysis of the supernatant by chromatography. The chromatographic conditions were as follows: mobile phase, liquid A, 0.04% formic acid aqueous solution and liquid B 0.04% formic acid acetonitrile solution; column temperature, 45 °C; flow rate, 400 µL per min; sample volume, 4 µL; chromatographic column, Waters, ACQUITY UPLCBEHC18 (2.1 × 100 mm, inner diameter 1.7 µm, Waters Corporation, Milford, MA, USA); electrospray ionization source; ion source temperature, 500 °C; Agilent 1290 HPLC-MS system (Santa Clara, CA, USA).

### 2.8. DNA Library Construction and Whole-Genome Bisulfite Sequencing

CK and D2 samples from before the last stress (CK-DB, D2-DB) were selected for methylation analysis. A Genomic DNA Extraction Kit (Tiangen Company, Huhhot, China, DP305) was used to extract genomic DNA from M. ruthenica leaf samples. DNA concentration, purity, and integrity were measured by spectrophotometry using NanoDrop or NanoPhotometer^®^ (IMPLEN, CA, USA) instruments and by agarose gel electrophoresis. DNA methylation analysis included one library per sample, and each library was sequenced separately. DNA libraries for heavy bisulfite sequencing were prepared as follows: genomic DNA was first sheared into 100–300 bp fragments by exposure to ultrasound (Covaris, MA, USA) and then purified with a MinElute PCR Purification Kit (QIAGEN, MD, USA). Then, DNA fragment end repair was conducted, an A base was added at the 3′ end, and the genomic DNA fragments were ligated to methylation sequencing adapters before bisulfite treatment using a ZYMO EZ DNA Methylation-Gold kit. Samples were then subjected to desalting and library fragment size selection. Finally, transformed DNA fragments were PCR amplified and sequenced using the Illumina HiSeqTM 2500 platform from Gene Denovo Biotechnology Co., Ltd. (Guangzhou, China); qualified libraries were selected for digital sequencing.

### 2.9. Genome-Wide Methylation Level Analysis

First, the reference genome was converted into a bisulfite version and then used as an index [29]. Sequence reads were also converted into complete bisulfite versions; then, they were aligned in a targeted manner with similar converted genome versions. Clean sequence reads were mapped to the *M. ruthenica* reference genome [24] (ASM1820801v1, ncbi.nlm.nih.gov/datasets/taxonomy/70973/, accessed on 1 August 2024) using BSMAP software [30] (version: 2.90) with default parameters. Methylated cytosine sites were identified by binomial test, which was applied to exclude non-conversion errors. Methylation levels were calculated based on methylated cytosine percentage in the whole genome, each chromosome, and different regions of the genome for each nucleotide sequence context (CG, CHG, and CHH).

Differential DNA methylation at each locus between the two experimental groups was determined using Pearson’s chi-square test (χ^2^) in methylKit [31] (version: 1.7.10). To identify differentially methylated cytosines (DMCs), the minimum read coverage to call the methylation status at a base was set to 4. A 200 bp window was used for whole genome scanning, the average DNA methylation rate was calculated within each window (for a certain type of C), and then we compared the differences in methylation levels among samples within each window. To identify differentially methylated regions (DMRs) between two samples, the minimum read coverage to call a methylation status at a base was set to 4.

Gene ontology (GO) enrichment analysis and Kyoto Encyclopedia of Genes and Genomes (KEGG) pathway enrichment analysis were conducted for DMC/DMR-related genes. GO enrichment analysis provides a comparison of all GO terms significantly enriched in genes within genomic backgrounds and filters out gene functions corresponding to biology. KEGG comprises publicly available molecular pathway-related data (http://www.kegg.jp/kegg/). Pathway enrichment analysis identified significantly enriched metabolic or signaling pathways in genes relative to the whole genome background.

### 2.10. RNA Extraction and Transcriptome Sequencing

Samples from plants in the CK and D2 groups collected before the final stress exposure (CK-DB, D2-DB) were selected for transcriptome analysis. Plant leaf RNA was extracted using a Total RNA Extraction Kit (TianGen Company, Huhhot, China, DP424). RNA concentration, purity, and integrity were measured using a spectrophotometer (NanoDrop). After total RNA extraction, eukaryotic mRNA was enriched with oligo (dT) beads; then, the enriched mRNA fragments were sheared into fragments using a fragment buffer and reverse transcribed into complementary DNA (cDNA) using an NEB Next Ultra RNA Library Prep Kit for Illumina. One transcriptome library was generated per sample, and each library was sequenced separately. Purified double-stranded cDNA fragments were end-filled; then, A bases were added, and they were ligated to Illumina sequencing adapters. Ligation reactions were purified using AMPure XP Beads (1.0×) and amplified by PCR. The resulting cDNA library was sequenced using the Illumina Novaseq 6000 System (Gene Denovo Biotechnology Co., Guangzhou, China).

### 2.11. Transcriptome Data Analysis

To obtain high-quality clean reads, reads were filtered using fastp [32] (version 0.18.0). The short reads alignment tool, Bowtie2 [29] (version 2.2.8), was used to map reads to an rRNA database. The rRNA mapped were then removed, and the remaining clean reads were further used for assembly and gene abundance calculation. An index of the reference genome and mapped clean reads complementary to the reference genome was established using HISAT2 (v2.4) [33]. FPKM values were calculated using RSEM (version 1.3.3) [34] software to quantify the expression abundance and variation in each transcription region. Edge R (version R-3.1) software was used to analyze the read count data obtained from the analysis of gene transcription levels in the CK and D2 groups, including the standardization of read counts and calculation of P and false discovery rate (FDR) values. FDR and differential multiple log2 fold-change (FC) values were used to screen for differentially expressed genes (DEGs) between the CK-DB and D2-DB groups; the default threshold was FDR < 0.05.

### 2.12. Quantitative Real-Time PCR Analysis

CK and D2 samples, including CK-DB, D2-DB, CK-DA, D2-DA, were analyzed to observe differences in gene expression. RNA concentration, purity, and integrity were measured using a spectrophotometer (NanoDrop). The National Center for Biotechnology Information (https://www.ncbi.nlm.nih.gov) was used to design primer sequences for RT-qPCR using sequence information obtained from transcriptome sequencing. Extracted RNA was used for cDNA synthesis with a reverse transcription kit (Takara, Beijing, China) and an RT-qPCR assay kit using a TB green mixture (Takara). Each experiment included three biological replicates and two technical replicates, and RT-qPCR reactions were conducted using an ABI real-time fluorescence quantitative PCR machine (QuantStudio 3, Thermo Fisher Scientific; Software, BioRad CFX). Values of ΔCT = CT target gene—CT actin and ΔΔCT = ΔCT(DS) − ΔCT(CK), relative to the CK group, were calculated to determine relative gene expression differences. After internal normalization, the relative expression differences of target genes are expressed as a 2^−ΔΔCT^ values, indicating the differential expression multiple of D2 plants relative to CK plants [27].

### 2.13. Statistical Analysis

SPSS statistical software version 26.0 (SPSS, Chicago, Illinois, USA) was used for analysis of variance and determining the significance of differences (*p* < 0.05).

## 3. Results

### 3.1. Phenotypic Changes Reveal the Response of M. ruthenica to Drought Stress

**Figure 2:**
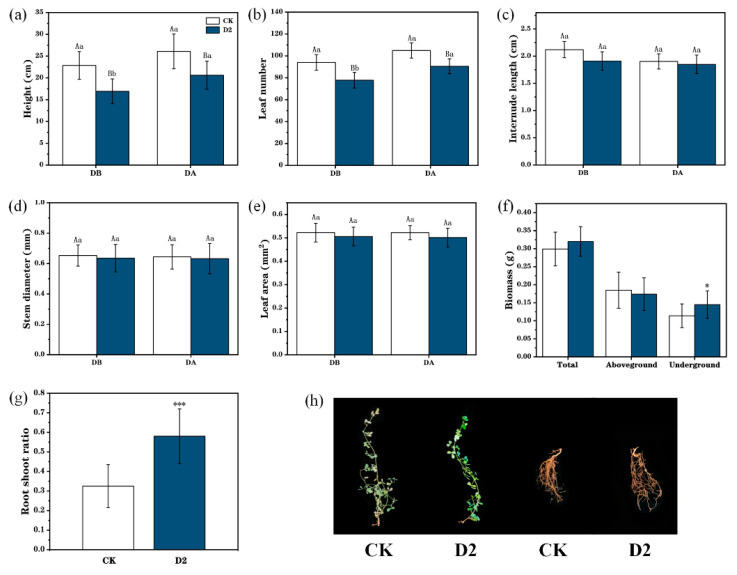
Differences in phenotypic traits between control (CK) and stress memory (D2) *M. ruthenica* before (DB) and after (DA) drought stress. (**a**) Height. (**b**) Leaf number. (**c**) Internode length. (**d**) Stem diameter. (**e**) Leaf area. (**f**) Biomass. (**g**) Root–shoot ratio. (**h**) “*”means *p* < 0.05; “***” means *p* < 0.001. Representative photographs showing phenotypic changes after the final drought stress. Uppercase letters indicate differences between different groups with the same treatment, and lowercase letters indicate the differences in the same group between treatments; error bars represent the standard error of the mean.

After exposure to two droughts periods, plant height and leaf number in the D2 group were significantly lower than those in the CK group (Figure 2a,b). During drought stress, CK and D2 plant height increased by 5.56% and 15.64% (Figure 2a), while the numbers of leaves in these two groups increased by 17.32% and 25.04%, respectively (Figure 2b). Further, the belowground biomass and root-to-shoot ratio were significantly higher in the D2 group than those of CK plants with increases of 27.19% and 78.46%, respectively (*p* < 0.05) (Figure 2f,g). In addition, the internode length (Figure 2c), stem diameter (Figure 2d), leaf area (Figure 2e), total biomass, and aboveground biomass (Figure 2f) were all decreased on drought exposure and did not differ significantly between the two groups. After the final drought stress, CK group leaves exhibited early wilting and yellowing, while those in the D2 group grew normally, and plant roots in group D2 were significantly larger than those in the CK group (Figure 2h).

### 3.2. Changes in Physiological Traits of M. ruthenica in Response to Drought Stress

**Figure 3:**
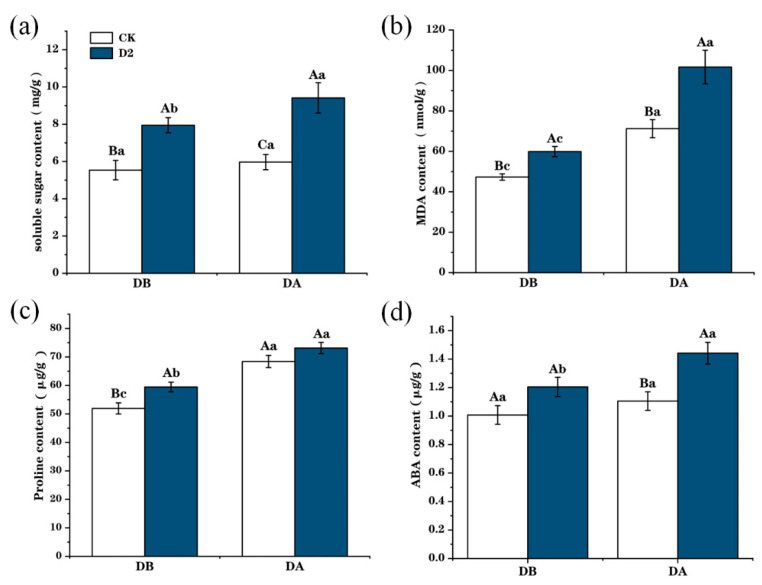
Changes in physiological indices of control (CK) and two drought-exposed (D2) *M. ruthenica* before (DB) and after (DA) drought stress. (**a**) Soluble sugar content. (**b**) MDA content. (**c**) Proline content. (**d**) ABA content. Uppercase letters indicate differences between different groups with the same treatment, and lowercase letters indicate the differences in the same group between treatments; error bars represent the standard error of the mean.

Drought stress led to increases in the content of soluble sugars, proline, MDA, and ABA in *M. ruthenica* leaves (Figure 3). Soluble sugar, proline, and MDA content were higher in the D2 group before the last drought stress (DB) than those in the CK group. In the D2 group, after the final drought stress (DA), the soluble sugar content was 1.58 times greater (Figure 3a) (*p* < 0.001), the MDA content was 1.43 times greater (Figure 3b) (*p* < 0.001), the proline content was 1.07 times greater (Figure 3c) (*p* < 0.05), and the ABA content was 1.25 times (Figure 3d) (*p* < 0.001) greater than those of the CK group plants.

### 3.3. Changes in DEGs of M. ruthenica under Drought Stress

We next analyzed transcriptome data from the CK and D2 groups before the last drought (CK-DB and D2-DB, respectively). Before the last drought stress, a total of 1495 DEGs were detected between the CK and D2 groups, including 969 up-regulated and 526 down-regulated genes (Supplementary Figure S1). GO enrichment analysis indicated that the 1495 DEGs were enriched in cell metabolism and environmental information processing among other pathways. In addition, KEGG analysis showed that DEGs were enriched in processes including arginine and proline metabolism, plant hormone signal transduction, and secondary metabolite synthesis.

### 3.4. Methylation Landscape of M. ruthenica under Drought Stress

**Figure 4:**
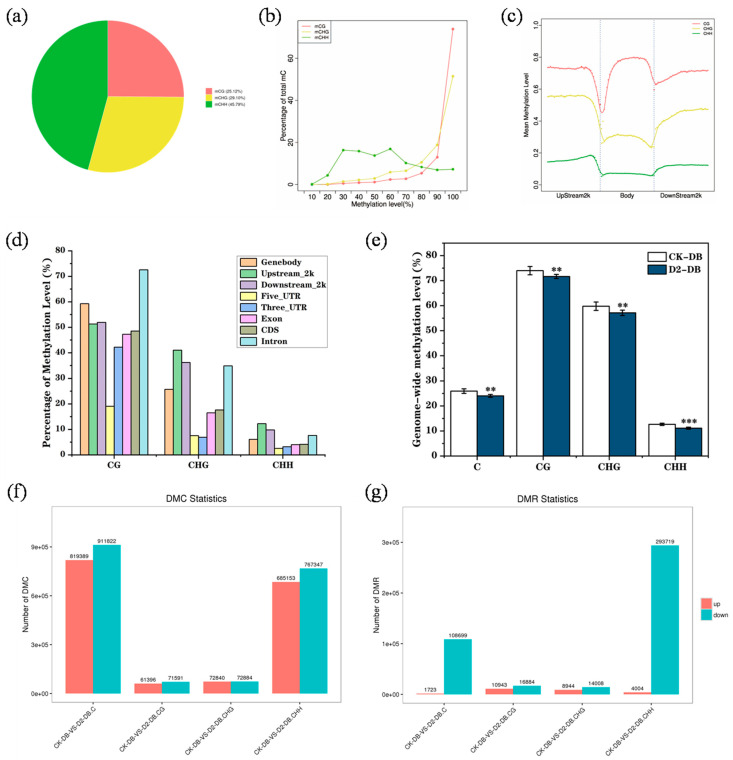
The *M. ruthenica* epigenome. (**a**) The relative proportion of methylcytosines (mCs) in three sequence contexts: CG, CHG, and CHH. (**b**) C-site methylation level distribution. (**c**,**d**) DNA methylation patterns in different genomic regions. (**e**) Whole-genome methylation levels in the CK and D2 groups. (**f**,**g**) Distribution of differentially methylated sites and regions in the CK and D2 groups.“**”means *p* < 0.01; “***” means *p* < 0.001.

We first analyzed the DNA methylation patterns in CK group *M. ruthenica* genomes. Methylcytosine was most commonly detected at CHH sites (45.79%) with fewer occurrences in CG and CHG contexts (25.12% and 29.10%, respectively) (Figure 4a). CHH methylation levels ranged from 10% to 90%, while those at CG and CHG sites were mainly > 70% (Figure 4b). Overall, the degree of methylation in gene body regions and at CG sites was highest (Figure 4c). In CG contexts, the methylation levels in intron and genomic regions were much higher than those in other regions. Methylation levels in upstream, downstream, and intron regions were highest in CHG contexts, and methylation levels in CHH contexts in various regions were similar to those in CHG contexts (Figure 4d).

We found that drought stress led to decreased methylation levels across the entire *M. ruthenica* genome (Figure 4e). A total of 819,289 up-regulated and 911,822 down-regulated methylation sites, as well as 1723 up-regulated methylation regions and 108,699 down-regulated methylation regions, were detected, and these changes mainly occurred in CHH contexts (Figure 4f,g).

### 3.5. Relationship between Methylation Levels and Gene Expression under Drought Stress

**Figure 5:**
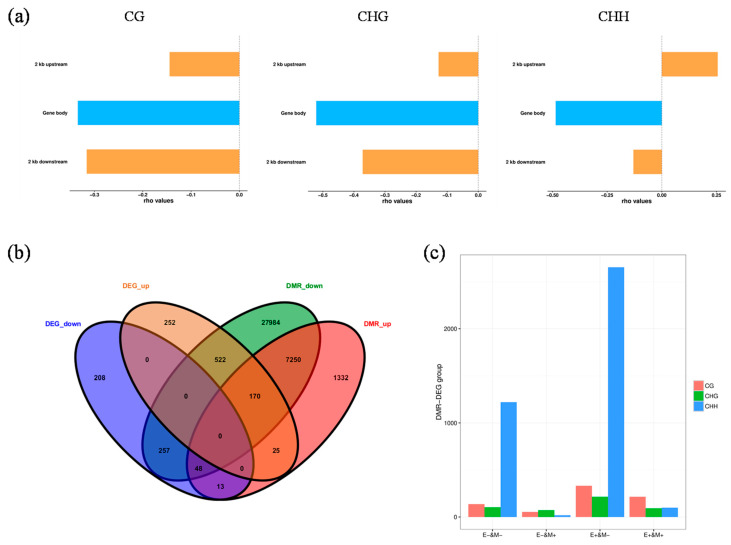
Analysis of correlations between methylation groups and transcriptome data in the D2 group. (**a**) Distribution plots of genes and DNA methylation levels with varying differential expression levels. The abscissa indicates the position from 2 kb upstream to 2 kb downstream; TSS, transcription start site; TTS, transcription termination site. The ordinate indicates the average methylation rate; special up, genes specifically up-regulated in the treatment group but not expressed in the control group; special down, genes specifically down-regulated in the control group but not expressed in the treatment group; other up, non-specifically up-regulated genes or genes up-regulated in the treatment group; other down, non-specifically down-regulated genes or genes down-regulated in the treatment group. (**b**) Venn diagram of differentially methylated regions (DMRs) and differentially expressed genes (DEGs). (**c**) Histogram showing changes in transcription levels of shared DMR-related genes and DEGs. E+/− indicates up-/down-regulation of gene transcription level. M+/− indicates up-/down-regulation of gene methylation level.

Focusing on the D2 group, which contained the most differentially expressed and methylated genes, we analyzed the relationships between DNA methylation and gene expression in different contexts. Within all three contexts analyzed, methylation levels in upstream promoter regions were high, which were followed by those in the downstream and gene regions. Methylation in CG and CHG contexts caused a considerable down-regulation of specific genes, while methylation in CHH sequences mainly caused non-specific gene down-regulation (Figure 5a,c). Analysis of correlations between DMRs and DEGs showed that more hypo-methylated genes had up-regulated transcription levels. Among overlapping DMRs and DEGs, 61 genes with down-regulated expression levels were hyper-methylated, and 692 with up-regulated expression levels were hypo-methylated in CK-DB vs. D2-DB; however, 195 up-regulated and 305 down-regulated genes were hyper-methylated and hypo-methylated, respectively (Figure 5b).

**Figure 6:**
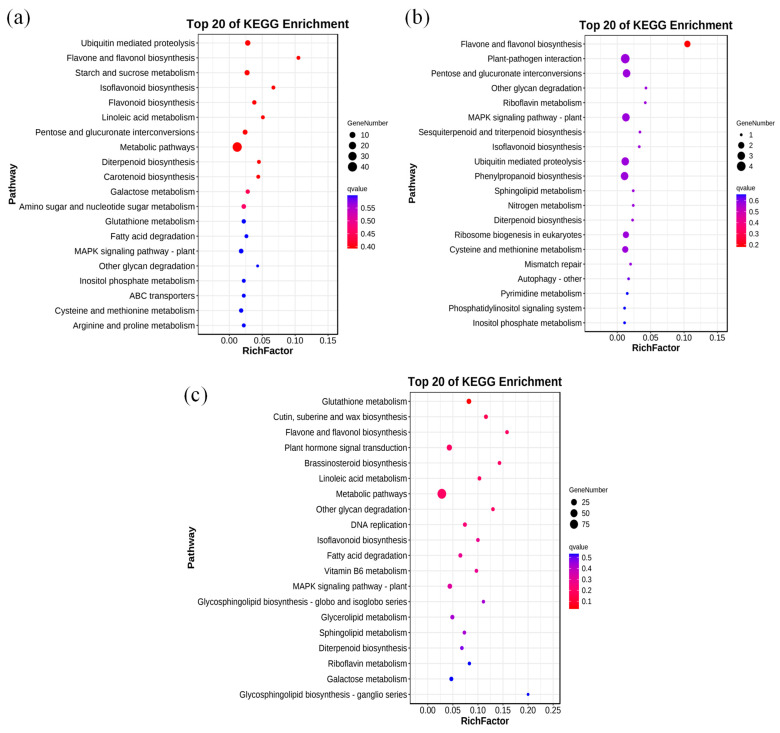
Enrichment analysis of differentially methylated region (DMR)- and differentially expressed gene (DEG)-related genes in three sequence contexts. Enrichment analysis of DMR- and DEG-related genes in (**a**) CG, (**b**) CHG, and (**c**) CHH contexts. Copyright permission has been granted for related KEGG images.

We next conducted an enrichment analysis of genes with associated DMRs and DEGs. Among the top 20 KEGG pathways, modifications occurred in a CHH context in 190 enriched genes, a CG context in 98 enriched genes, and a CHG context in 34 enriched genes (Figure 6). CG context methylations were mainly enriched in metabolic pathways, protein proteolysis, and starch sucrose metabolism. Notably, carotenoid synthesis and proline metabolism pathways were also enriched in CG context methylations (Figure 6a). CHG context methylations were in genes enriched in pathways including flavonoid and flavonoid biosynthesis (Figure 6b). CHH context methylations were in genes enriched in metabolic pathways, plant hormone signal transduction, glutathione metabolism, and cutin suberin and wax biosynthesis (Figure 6c).

**Figure 7:**
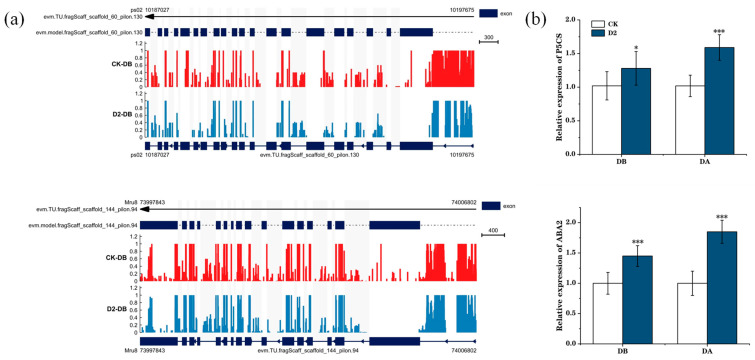
Visualization of methylation and relative expression levels of *P5CS* and *ABA2* before and after the final drought stress. (**a**) IGV(version 2.16.0) software analysis of stress-induced demethylation of the *P5CS* and *ABA2* promoter and gene regions. (**b**) Relative expression levels of *P5CS* and *ABA2*. “*” means *p* < 0.05; “***” means *p* < 0.001.

**Figure 8:**
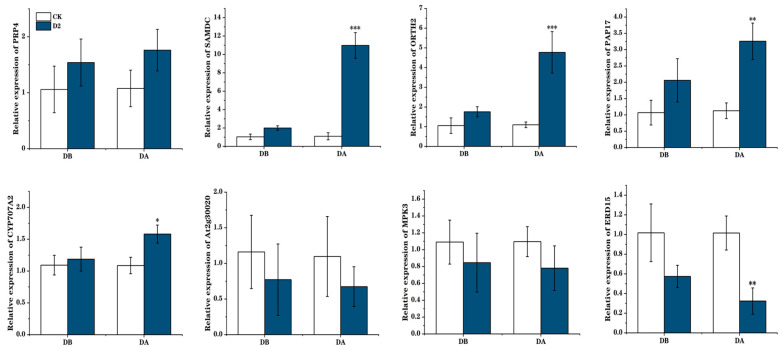
Validation of gene expression levels under drought stress by real-time quantitative PCR. Bars represent mean ± SD values from three biological replicates. “*” means *p* < 0.05; “**” means *p* < 0.01; “***” means *p* < 0.001.

We next focused on the proline and ABA biosynthetic pathways and ultimately on two genes: *ABA2*, encoding the enzyme, zeaxanthin epoxidase, which is involved in carotenoid synthesis, and *P5CS*, encoding an enzyme involved in proline synthesis, Δ1-pyrroline-5-carboxylate synthase. Visualization of the methylation levels at these two genes illustrated hypo-methylation of the gene and promoter regions in the D2 group, but not in the CK group, before the drought. We also observed the relative expression levels of these two genes before and after the final drought. In the D2 group, the expression levels before drought were higher than those in the CK group, and they rose significantly after drought (*p* < 0.001) (Figure 7). Many other important genes were also identified, including *PRP4* and *SAMDC*, which are involved in arginine and proline metabolism; *CYP707A2*, *At2g30020*, and *MPK3*, involved in ABA biosynthesis; *ERD15*, involved in dehydration stress response; and *DRTH2*, involved in DNA methylation; indicating that these genes have important roles in regulating plant drought stress memory (Figure 8).

## 4. Discussion

### 4.1. M. ruthenica Plants Actively Respond to Drought Stress, Due to Stress Memory

Stress memory, when plants store and retain previous stress cues and exhibit stronger and faster responses to repetitive events, is regulated by various mechanisms [27,35]. Drought, high temperature, and salt alkali stress can induce stress memory, helping plants cope with these stresses [36].

In this study, we found that *M. ruthenica* can develop stress memory after experiencing one or two drought exposures and that two drought exposures were more likely to help plants develop stress memory. Therefore, we chose D2 for use in subsequent analyses. Stress memory can help plants respond more actively to subsequent drought stress, and it is mainly manifested as changes in phenotypic responses and physiological indicators. Our results support a role for stress memory and drought tolerance in enhancing plant biomass. The aboveground leaves of plants in the D2 group exhibited less wilting, while their underground biomass was significantly higher than that of CK group plants. Further, total biomass was also higher in D2 group plants (Figure 1 and Figure 2), which may be attributable to epigenetics-mediated mechanisms underlying stress memory. DNA methylation ‘priming’ in D2 plants does not function by increasing their biomass, but it rather increases the flexibility of plants to respond to environmental stress, resulting in stronger resistance to drought stress [9,37,38].

### 4.2. DNA Methylation May Contribute to Plants Stress Memory Formation

Plants can undergo epigenetic changes to adapt to stress, and such epigenetic alterations are more flexible than genetic variation. A proportion of epigenetic changes are temporary and can be restored, some of which are heritable, and this is referred to as epigenetic memory [6,39,40,41]. DNA methylation is among the main epigenetic modifications commonly found in eukaryotic genomes. Methylation groups vary among different plants, as do changes in methylation groups after drought stress [42,43]. In our study, genomic DNA methylation levels were highest at CG sites (>70%), intermediate at CHG sites (60%–65%), and lowest at CHH sites (<15%), which is consistent with findings in *Arabidopsis* [44,45]. Methylcytosine was most commonly detected at CHH sites (45.79%), and DMRs were also most abundant in CHH contexts [46], indicating that CHH contexts are more sensitive to drought stress in *M. ruthenica*. Hyper-methylation/hypo-methylation at CHH sites may constitute a new epigenetic modification that regulates the growth performance of higher plants under stress [47]. Cytosine methylation levels in CG, CHG, and CHH contexts can affect gene expression as well as having crucial roles in plant drought stress responses [37,48]. Changes in DNA methylation vary among different plants under drought stress; generally, drought-tolerant plants have more stable methyl groups under drought conditions, manifesting as low methylation levels [38,49]. We found that *M. ruthenica* methylation groups were relatively stable, while drought stress caused a slight decrease in DNA methylation; similar results have been reported in *Medicago sativa*, *Lolium perenne*, and *rice* [38,50,51,52]. DNA methylation is established to play an important role in plant responses to drought stress, and it may constitute plant drought stress memory. Drought stress triggers epigenetic markers in plants, including DNA methylation, which lead to changes in gene expression, signaling pathways, and phenotypic modifications, ultimately improving adaptation to environmental changes [6,40,53,54]. In rice , 70% of drought-induced epigenetic methylation sites are demethylated, and stress exposure during the reproductive stage leads to a negative correlation between yield and methylation rate, indicating critical crosstalk between epigenetic and reproductive signals in rice plants under drought stress [55,56]. DNA methylation in wild strawberries (*Fragaria nilgerrensis*) under drought stress may affect gene expression, regulating osmotic capacity and maintaining a balance between reactive oxygen species regeneration and clearance through ABA-dependent signaling pathways, affecting plant drought tolerance [57]. In this study, we found that *M. ruthenica* genomic methylation levels decreased after two drought stress exposures, which may have triggered ‘priming’ stress mechanisms in the plants. Therefore, when plants were subsequently exposed to drought stress, the drought stress memory was quickly activated, helping the plant make changes to more rapidly adapt to stress.

### 4.3. Relationship between DNA Methylation and Gene Expression

Most often, the presence of methylation does not influence gene transcription; however, under environmental stress conditions, changes in DNA methylation may alter gene expression, leading to visible phenotypes [58]. The regulatory effect of DNA methylation on plant gene expression has been widely studied, and numerous investigations have shown that correlations between DNA methylation and gene expression are very subtle, with changes in DNA methylation status in different regions (upstream, gene body, and downstream) leading to differences in gene expression. In rice, promoter DNA methylation is associated with down-regulated genes, while genomic methylation typically promotes transcription [59]. In apple genomes, the DNA methylation of gene bodies is positively correlated with gene expression, while there is no significant correlation between promoter region methylation and gene expression [58,60]. Interestingly, there are positive and negative correlations between gene expression and methylation in the promoter and genome regions, respectively, of wild strawberries [57,61]. Here, we found that changes in promoter region methylation levels had greater impacts on gene expression variation, and methylation levels in promoter regions were highest in all three nucleotide contexts examined, which can lead to a down-regulation of gene expression, particularly in CG and CHG contexts; numerous genes that were specifically down-regulated encoded key factors involved in drought stress memory response. In addition, we identified 38,061 differential methylation and expression-related genes, which showed significant differences in both methylation and expression levels under drought stress, indicating a significant correlation between DNA methylation and gene transcription; however, our data also indicate that there are multiple types of association between DNA methylation and gene expression rather than a simple linear correlation. The number of genes up-regulated by demethylation was highest, indicating that demethylation may have an important role in drought stress and a potential regulatory effect of DNA methylation on gene expression [47].

### 4.4. The Role of Memory Genes in Maintaining M. ruthenica Drought Stress Memory

Memory genes contribute to initial drought stress exposure responses, and changes in their transcription levels during subsequent stress may allow plants to fine-tune their response to sustained drought stress [62]. Transcriptional memory can provide the benefits of stronger or modified stress responses while reducing the cost of coping with stress [11,63].

Proline is an amino acid synthesized in higher plants that has an important role in response to drought stress [42,64]. After experiencing drought stress, plants can generate more proline to eliminate stress-mediated harm [65]. Our results indicate that drought stress leads to an increase in proline content, which is consistent with the findings of previous studies [24]. Δ1-pyrroline-5-carboxylate synthase (*P5CS*) is a key enzyme in the proline synthesis pathway, catalyzing the conversion of glutamic acid to the intermediate compound, glutamic acid-γ-semialdehyde [66]. The accumulation of high levels of proline and an elevated expression of *P5CS* in D2 group plants during the final drought stress in our experiment may be attributable to the effect of drought stress memory. Proline accumulation was also observed in rice after multiple drought stress exposures, which is similar to our findings [67]. In addition, increased proline induced by salt stress also exhibits a memory-related pattern, which is similar to the drought stress memory training observed in our study [68]. In both *maize* and *Arabidopsis*, many genes involved in proline synthesis and metabolism contribute to plant drought stress responses, and these are defined as memory genes [11,16,24]; for example, in rice, *P5CS1* is involved in proline biosynthesis and exhibits transcriptional memory after repeated drought stress, indicating a potential role in drought tolerance [3,67].

ABA is among the most important plant stress hormones and is involved in various important physiological processes throughout the plant life cycle, including stress response, development, and reproduction [69,70,71]. Arabidopsis responds to repeated dehydration stress by accumulating ABA at concentrations two to three times those in controls [72]. We observed a similar phenomenon, where the ABA content in M. ruthenica leaves was considerably higher after exposure to two droughts than that in the control group, suggesting that ABA may be involved in drought stress memory in this plant. The transcript levels of key regulatory genes in the Arabidopsis ABA biosynthesis pathway were increased, and ABA biosynthesis was active in response to drought [71,73,74]. *ABA2* participates in the final step of ABA biosynthesis and can catalyze the conversion of flavin to abscisic aldehyde, playing a significant role in the regulation of plant endogenous ABA levels [75,76]. We also observed a demethylation of the *ABA2* gene and promoter regions in the D2 group, and its gene expression levels also showed transcriptional memory. Therefore, we speculate that the *ABA2* catalysis of ABA synthesis contributes to the regulation of drought stress memory in *M. ruthenica*.

## 5. Conclusions

**Figure 9:**
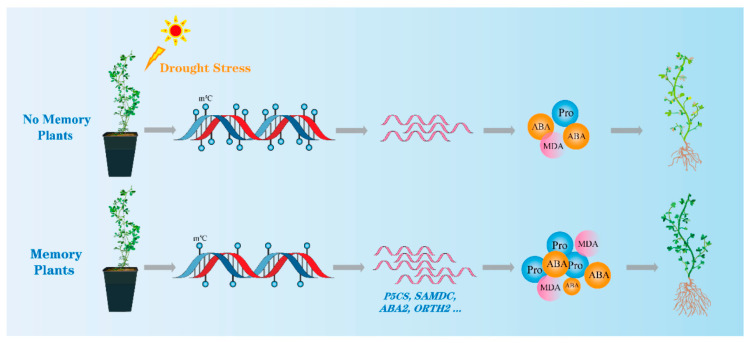
Conceptual diagram of drought stress memory in *M. ruthenica*.

Our genome-wide methylation and transcriptome analysis data reveal different methylation regions and genes that are potentially altered under drought stress, providing a reference for the future breeding of alfalfa species. These results indicate that *M. ruthenica* plants can generate short-term stress memory after experiencing drought stress, and that this stress memory can help plants actively cope with subsequent drought stress exposure. Epigenetic modifications, such as the down-regulation of genomic methylation levels, may be a mechanism underlying stress memory formation. Decreased gene methylation levels can alter gene expression levels and affect drought response signaling, such as the ABA and proline synthesis pathways in plants, facilitating a more rapid response to drought stress and improving drought tolerance (Figure 9). These findings expand our understanding of the mechanisms underlying short-term drought stress memory in plants, which help them adapt to environmental changes.

## Data Availability

The *M. ruthenica* line used in this experiment was provided by the Institute of Grassland Research of CAAS. All materials including all relevant raw data described in the manuscript are available upon request. The datasets generated and analyzed during the current study are available in the online repository: https://www.ncbi.nlm.nih.gov/sra/?term=PRJNA1039991 (accessed on 1 August 2024).

## Supplementary Materials

The following supporting information can be downloaded at: https://www.mdpi.com/article/10.3390/genes15101286/s1, Table S1. qRT-PCR primers of genes related to the drought stress memory in *M. ruthenica* leaves. Figure S1. Differences between CK and D2 plants at the transcriptome level. Figure S2. Transcriptome sequencing FPKM of drought stress memory regulatory genes in *M. ruthenica*.
