# Supplementary material for: DNA Methylation Participates in Drought Stress Memory and Response to Drought in Medicago ruthenica
Source: Genes (Basel). 2024 Sep 30;15(10):1286. doi: 10.3390/genes15101286 (PMC11507442; doi:10.3390/genes15101286)
Supplement: Supplementary file 1 [file genes-15-01286-s001.zip › genes-3164334-supplementary.pdf]

Supplementary Material

Table S1 qRT-PCR primers of genes related to the drought stress memory in *M. ruthenica* leaves

Gene	Prime NO.	Primer Sequence（5'→3'）	Product size(bp)
Actin	Actin-F	ATCCAGGCTGTCCTCTCCCT	20
	Actin-R	ACGAAGGATGGCATGTGGGA	20
P5CS	P5CS-F	TGGTGGACCAAAAGCTAGTGT	21
	P5CS-R	TGTGGTCAATCGCAGCAAAT	20
ABA2	ABA2-F	GGAAGTGCAGTGCAAGAAGAT	21
	ABA2-R	CCAAGTGCCATGTTCACTCC	20
PRP4	PRP4-F	TCCTCCTCCTGTTCCAGTCA	20
	PRP4-r	GTGGTTGTGGCTTTGGAACT	20
SAMDC	SAMDC-F	TGCATCTTCAGCAGGTTTGA	20
	SAMDC-R	TTGAGTAGCCGCAAGGTTCA	20
ORTH2	ORTH2-F	CGGTGGAAGGGATCTCAGTG	20
	ORTH2-R	CAACTGGTTCGCAAAGCCTC	20
PAP17	PAP17-F	GGTGGCTTTGTTTAAGGTCTTTCA	24
	PAP17-R	CAACCAGCTCTGCACAAGAAC	21
CYP707A2	CYP707A2-F	TGGTTCAATGGGTTGGCCTT	20

	CYP707A2-R	ATATCGACGTGAGCATGTGT	20
At2g30020	At2g30020-F	CTGGTGGTGATGCTTGTGGT	20
	At2g30020-R	GGAGATCTGAAGAGAAAGGAGTGA	24
MPK3	MPK3-F	CACGAGATGGTATAGGGCTCC	21
	MPK3-R	TGCCAGGAAACAGAGGCTTT	20
MtERD15	MtERD15-F	TTTTCTCCGGAATGGTGGCA	20
	MtERD15-R	TCGTCATCATGTTGCTGGCT	20

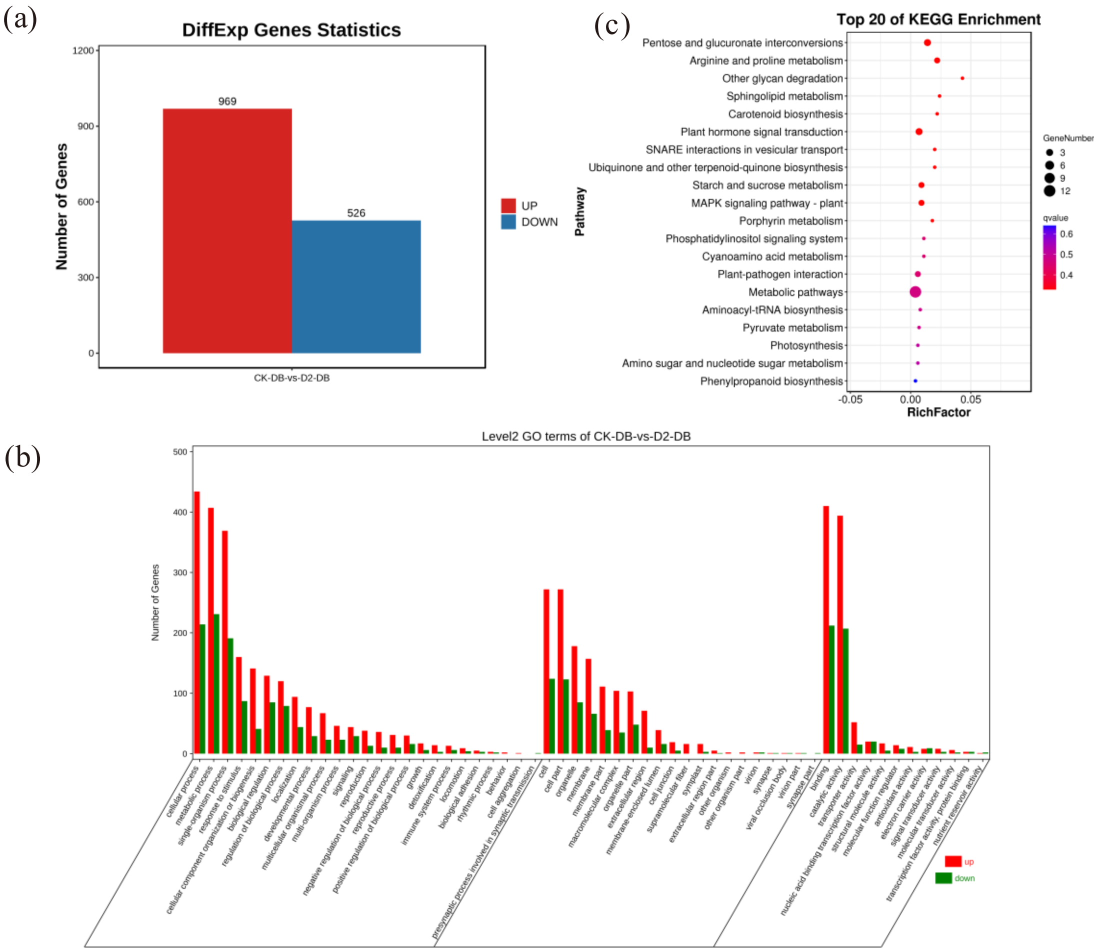

Figure S1. Differences between CK and D2 plants at the transcriptome level. (a) Number of differentially expressed genes (DEGs) between the CK and D2 groups. (b) Gene Ontology (GO) enrichment map of DEGs. (c) Kyoto Encyclopedia of Genes and Genomes (KEGG) DEGs enrichment map.

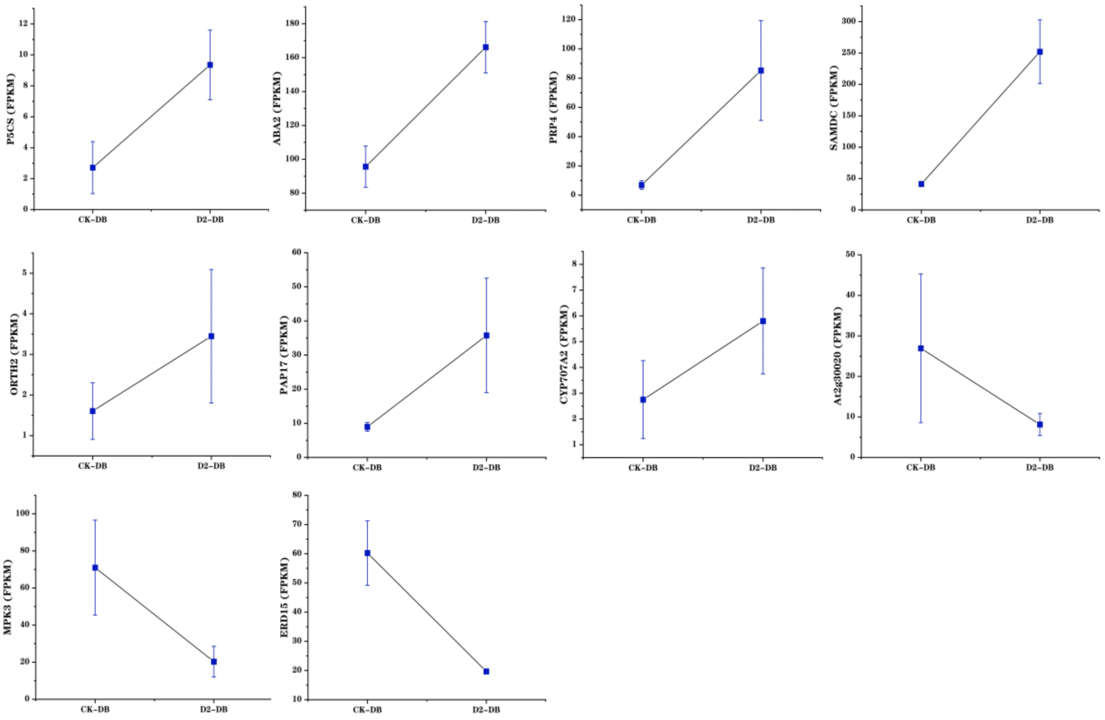

Figure S2. Transcriptome sequencing FPKM of drought stress memory regulatory genes in *M. ruthenica*.
